# Barriers and enhancers to COVID-19 vaccination among healthcare workers in a metropolitan city in Nigeria

**DOI:** 10.4102/jphia.v16i1.685

**Published:** 2025-04-28

**Authors:** Adewale V. Opayele, Adeniyi F. Fagbamigbe, Chinwe L. Ochu, Rodgers R. Ayebare, Adedayo O. Faneye, Adewemimo C. Olaosebikan, Oluwaseun E. Falayi, Gloria O. Nwiyi, Sunday O. Eziechina, Ikemefule R. Uzoma, Priscilla Ibekwe, Prosper Okonkwo, Tamrat Shaweno, Nebiyu Dereje, Suzan Nakasendwa, Tonny Muwonge, Mosoka P. Fallah, Georgina N. Odaibo

**Affiliations:** 1 Department of Virology, College of Medicine, University of Ibadan, Ibadan, Nigeria; 2 Department of Epidemiology and Medical Statistics, Faculty of Public Health, University of Ibadan, Ibadan, Nigeria; 3 Nigeria Centres for Disease Control and Prevention, Abuja, Nigeria; 4 Infectious Diseases Institute, Makerere University, Makere, Uganda; 5 Department of Epidemiology and Medical Statistics, College of Medicine, University of Ibadan, Ibadan, Nigeria; 6 APIN Public Health Initiative, Abuja, Nigeria; 7 Africa Centres for Disease Control and Prevention, Addis Abada, Ethiopia

**Keywords:** Ibadan, vaccine uptake, vaccine acceptance, vaccine hesitancy, coronavirus

## Abstract

**Background:**

Vaccine hesitancy among healthcare workers (HCWs) hinders coronavirus disease 2019 (COVID-19) control efforts.

**Aim:**

The aim of the study was to assess enhancers and barriers to the uptake of COVID-19 vaccine among HCWs in Ibadan, Nigeria.

**Setting:**

Health facility-based cross-sectional study in Ibadan, a metropolitan city in Oyo state, Nigeria.

**Methods:**

A questionnaire administered using REDCap assessed HCW vaccination status, attitudes and access using the Behavioural and Social drivers (BeSD) framework. Data analysis performed using STATA version 17 included descriptive statistics and modified Poisson regression.

**Results:**

Of the 1227 HCWs recruited, 82.8% received at least one dose. Vaccine uptake was higher among older HCWs compared to those below 25 years (45–54 years: prevalence ratio [PR] = 1.20, 95% confidence interval [CI]: 1.09, 1.33 and ≥ 55 years: PR = 1.17, 95% CI: 1.05, 1.30) and HCWs in private health centres (PR = 1.15, 95% CI: 1.08, 1.22). Most HCWs (83.5%) believed in vaccination for infectious diseases, but only 61.9% felt the same about COVID-19 vaccines. The major reasons for vaccine hesitancy among unvaccinated HCWs included the beliefs that vaccine development and authorisation were rushed (47 [26.1%]) and concerns about serious side effects (32 [17.8%]).

**Conclusion:**

This study found that the uncertainty about COVID-19 vaccine safety is a key barrier to its uptake. Therefore, targeted education and communication strategies to improve vaccine confidence are crucial.

**Contribution:**

This study identifies why HCWs in Nigeria are hesitant about getting vaccinated. This information can help to improve vaccination rates in this group. It fits with the journal’s focus on making African public health responses stronger.

## Introduction

### Background

In December 2019, the first case of a novel coronavirus was reported in Wuhan, China. This virus later spread to cause the COVID-19 pandemic, which the World Health Organization (WHO) declared a Public Health Emergency of International Concern (PHEIC) on 11 March 2020. As of 11 February 2024, the total reported cases was 774 631 444.^1^ The devastating impact of this outbreak was felt globally until control efforts started yielding positive outcomes, including the development of vaccines. However, because of vaccine manufacturers’ inability to meet global demands at the beginning of vaccination campaigns in Africa, priority was given to health workers, teachers and the elderly.^2,3^ Subsequently, efforts towards global vaccine equity increased the supply of vaccines across Africa. This mission was achieved through the intervention of programmes such as the Saving Lives and Livelihoods (SLL), a joint initiative with the Africa Union, Africa Centers for Disease Control and Prevention (CDC) and the Mastercard Foundation.^4^ It supported purchasing and delivering COVID-19 vaccines across the five African Union regions. Other supporting partners in this effort include the Africa Vaccine Acquisition Trust (AVAT), COVID-19 Vaccines Global Access (COVAX), the WHO, the United Nations Children’s Fund (UNICEF) and the governments of various countries.^5^ Despite this massive intervention, vaccine uptake remains low in many African countries, which can be traced to vaccine hesitancy among the populace.^6^

Even though the various available COVID-19 vaccines have been proven to be efficacious and safe,^7^ many individuals prefer to remain unvaccinated because of prevailing misconceptions about the vaccines.^8^ vaccine hesitancy, manifested as a delay in acceptance or outright refusal of vaccine services, can fuel the spread of the virus among a population. Hesitancy develops when there is a low perception of the need for a vaccine, concerns over the efficacy and safety of the vaccine and consideration of ease of accessing the vaccine.^9^ The determinants of vaccine hesitancy are complex and fuelled in part by misinformation or limited and controversial information, sociocultural factors, increasing individuals’ perceptions of their right to refuse medical services and decreasing trust in governmental institutions.^10,11^ Although the gains and cost-effectiveness of vaccines are undisputable, the individual and collective benefits they achieve are contingent on the behaviours and attitudes of individuals.

Studies have shown that healthcare workers (HCWs) play a crucial role in influencing vaccine acceptance among the general population. They are often perceived as the most reliable source of health-related advice, including vaccination and are at the forefront of delivering vaccines and other healthcare services. Therefore, their inner conviction about vaccines and commitment to advocating their acceptance to the community members they serve is vital to the success of any vaccination programme. Thus, if HCWs have personal reservations about specific vaccines, this can impede their promotion and uptake among the populace.^12^ Furthermore, numerous studies have shown that HCWs are more likely to promote vaccination to patients if they have been vaccinated themselves.^13,14^

Intention to accept COVID-19 vaccination varies among HCWs in different African countries, with reported rates of 27.7% in the Democratic Republic of the Congo,^15^ 39.3% in Ghana^16^ and 63.8% in Sudan.^17^ In Nigeria, information is scarce on such intentions among HCWs. However, rates of 34.7% and 40.0% have been reported among staff and students in tertiary institutions in the Southeastern and North-Western parts of the country, respectively.^18,19^ The HCWs in Nigeria have also been reported to have more awareness of COVID-19 vaccines than members of the general adult populace.^20^ It’s important to notice that some individuals who express an intention to get vaccinated may ultimately not follow through with getting vaccinated against COVID-19. This discrepancy can be attributed to several factors, including vaccine availability, ease of accessibility and several other individual circumstances.^21^ This study was, therefore, designed to determine the actual vaccination uptake and identify the factors that serve as enhancers or barriers to COVID-19 vaccination among HCWs in Ibadan, a metropolitan city in Oyo state, Nigeria.

## Research methods and design

### Study design

This was a cross-sectional study conducted from April 2023 to May 2023.

### Study setting

**FIGURE 1:**
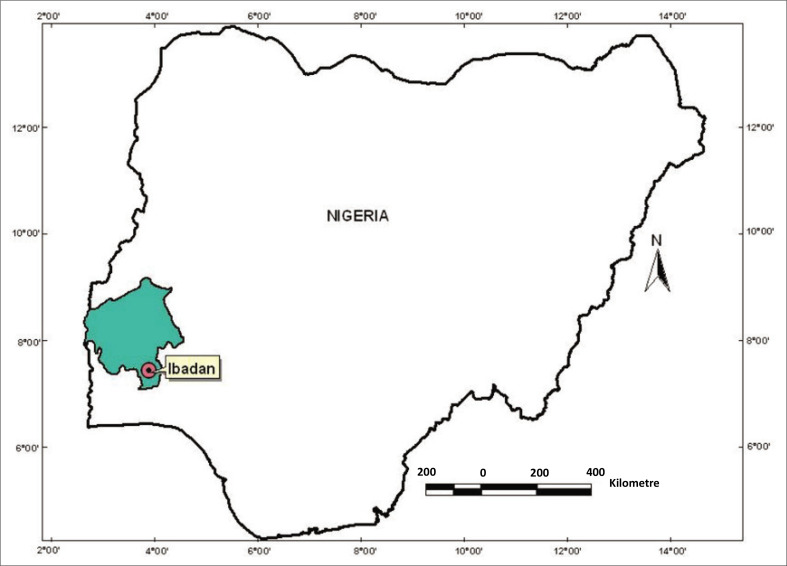
Map of the study location in Ibadan, Oyo state (southwest geopolitical zone), Nigeria.

The study was conducted in Ibadan, a metropolitan city in Oyo state, Nigeria. Ibadan has a population of 3.6 million and was considered a COVID-19 hotspot with low vaccination rates. The city was identified as one of the hotspots for the COVID-19 epidemic in Nigeria, with COVID-19 vaccination rates falling below the WHO and Africa CDC target of 70% by June 2022. As of April 2023, over 10 400 confirmed cases of COVID-19 had been reported in Oyo state. Figure 1 is a map of Nigeria showing the locations of Oyo state and Ibadan. Ibadan is a metropolitan city comprising 11 of the 33 Local Government Areas (LGAs) in Oyo state. A total of 28 health facilities (primary, secondary, tertiary, public and private) were included.

### Study population

The study population consisted of HCWs from various professions including physicians, nurses, midwives, pharmacists, laboratory personnel, community health workers, radiographers, hospital ward assistants and cleaners.

### Sampling strategy

A purposive sample of 1227 HCWs was recruited. The sample aimed for representation across cadres to ensure a diverse group of participants.

#### Inclusion criteria

The HCWs currently practicing in any healthcare facility in Ibadan were included in this study.

#### Exclusion criteria

The HCWs not currently practicing in healthcare were excluded in this study.

### Data collection

Data were collected using an interviewer-administered survey instrument developed based on the Behavioural and Social drivers (BeSD) framework for vaccine demand creation.^22^ The questionnaire was administered electronically via REDCap by trained research assistants.

### Data analysis

Data were analysed using STATA version 17. Descriptive statistics summarised participant demographics and vaccine attitudes. Bivariate analysis (Chi-square) examined associations between variables. A modified Poisson regression model identified factors associated with vaccination status. Adjusted prevalence ratios (aPR) with 95% confidence intervals (CI) were used to assess these associations. Statistical significance was set at *p* < 0.05.

### Ethical considerations

Ethical clearance to conduct this study was obtained from the Oyo State Ministry of Health Ethics Committee (No. NHREC/OYOSHRIEC/10/11/22). All study participants gave written informed consent prior to the interviews. Investigators and research assistants completed the National Institute on Drug Abuse (NIDA) online course on good clinical practice.

## Results

### Demographics of research participants

Most of the 1227 HCWs enrolled were females 833 (67.9%), while the largest proportion were in the 45 years and above age group 267 (31.7%). Majority of participants worked in public healthcare facilities, 1091 (88.9%), with most health facilities located in urban areas. Of all the health professionals enrolled, the largest proportions (28.5%) were nurses and midwives, while the least was community support (9.3%). The demographic characteristics of the study participants are presented in Table 1.

**TABLE 1:** Demographic characteristics among health care workers by willingness to recommend vaccination in Oyo State.

Variables	Frequency (*n*)	%	Definitely/probably recommend		Definitely/probably not recommend		*P*
			*n*	%	*n*	%	
**Gender**	-	-	-	-	-	-	0.03*
Male	394	32.1	355	90.1	39	9.9	-
Female	833	67.9	780	93.6	53	6.4	-
**Age (years)**	-	-	-	-	-	-	0.39
18–24	153	12.5	141	92.2	12	7.8	-
25–34	371	30.2	337	90.8	34	9.2	-
35–44	314	25.6	289	92.0	25	8.0	-
45–54	267	21.8	253	94.8	14	5.2	-
55+	122	9.9	115	94.3	7	5.7	-
**Location**	-	-	-	-	-	-	0.67
Urban	1207	98.4	1117	92.5	90	7.5	-
Peri-urban	20	1.6	18	90.0	2	10.0	-
**Job designation (professions)**	-	-	-	-	-	-	0.10
Physicians	268	21.8	247	92.2	21	7.8	-
Nursing and midwifery	350	28.5	331	94.6	19	5.4	-
Pharmaceutical person	85	6.9	77	90.6	8	9.4	-
Laboratory health worker	256	20.9	233	91.0	23	9.0	-
Community support	114	9.3	147	95.5	7	4.6	-
Other health workers	154	12.6	100	87.7	14	12.3	-
**Facility ownership**	-	-	-	-	-	-	0.03*
Public	1091	88.9	1003	91.9	59	5.4	-
Private	136	11.1	132	97.1	3	2.2	-

**Total**	**1227**	**100**	**1135**	**92.4**	**92**	**7.6**	**-**

Note: Job designation ‘Other HCWs’ include hospital cleaners, ward maids, radiographers and so on.

HCW, healthcare workers.

* , Represent statistically significant variables at 0.05.

### Vaccination uptake among healthcare workers in Ibadan

**FIGURE 2:**
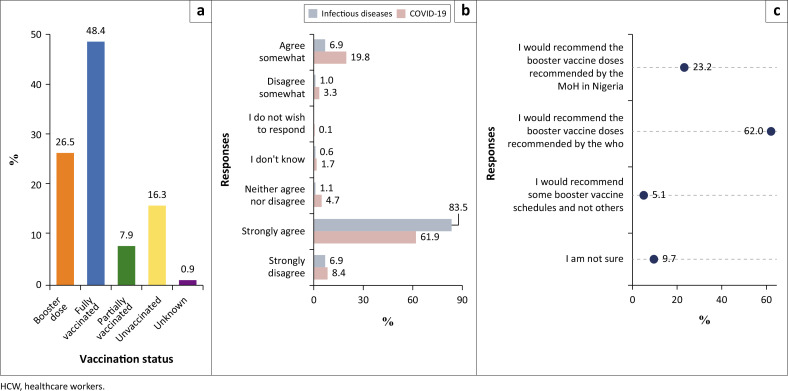
Combination of figures from the study. (a) Level of vaccine update among HCWs in Ibadan, Oyo state, Nigeria; (b) Confidence in the effectiveness of vaccines among HCWs in Ibadan, Oyo state; and (c) HCWs willingness to recommend COVID-19 boosters vaccine doses based on source of information.

A large proportion of the participants (74.9%) had received all doses of their vaccine types, including 325 (26.5%) who had received a booster dose. Only 200 (16.3%) of the 1227 participants were unvaccinated (Figure 2a).

### Access to COVID-19 vaccines among healthcare workers in Ibadan

Among the HCWs vaccinated against COVID-19, only 5.5% reported that accessing the vaccine was ‘Not at all easy’, while 12.4% found it ‘A little easy’. Followed by 23.4% who reported that accessing the vaccine was ‘Moderately easy’. Majority of the HCWs (58.7%) deemed it ‘Very easy’ to access COVID-19 vaccines.

### Confidence in the ability to answer COVID-19-related questions

Only 29 of the 1227 participants (2.4%) admitted to being ‘Not at all confident’ in their capacity to address inquiries concerning COVID-19. Whereas 14.6%, 27.8% and 52.8% indicated that they were ‘A little confident’, ‘Moderately confident’ and ‘Very confident’, respectively, in their capacity to effectively address patient queries concerning COVID-19. A small proportion (2.4%) expressed uncertainty or ‘unsure’ about their confidence.

### Vaccination status and factors associated with the vaccine uptake

Vaccination uptake was higher in primary (88%) than in secondary (84%) and tertiary (79%) health facilities. The association between the facility level and vaccine uptake was significant (*p* = 0.012).

As shown in Table 2, age was a statistically significant factor influencing vaccination status (*p* < 0.01). The participants aged 45 years and above had the highest vaccination rate of 90.2% (aPR: 1.20; 95% CI: 1.09–1.33) and aPR = 1.17 (95% CI: 1.05–1.30) for 45–54 years and > 55 years and above, respectively). The job role of HCWs also exhibited a statistical association with vaccination status. For instance, vaccination status was 15% higher among physicians than the pharmaceutical personnel (aPR = 1.15; 95% CI: 1.02–1.31; *p* < 0.01). Healthcare workers categorised as ‘community support workers’ and ‘others’ had the highest vaccination uptake of 91.6% and 90.2%, respectively, while the lowest uptake was among laboratory HCWs (68.9%). Facility ownership also had a substantial effect on vaccination status with HCWs in private healthcare facilities having a higher (93.4%) vaccination uptake compared to public healthcare facilities (*p* < 0.01).

**TABLE 2:** Factors associated with vaccination status among healthcare workers in Ibadan.

Variables	Unvaccinated		Vaccinated		Total	*P*	Adjusted prevalence ratio	95% CI
	*n*	%	*n*	%				
**Gender**	-	-	-	-	-	< 0.974	-	-
Male	65	16.5	329	83.5	394	-	1.00	-
Female	135	16.4	687	83.6	822	-	1.01	0.95–1.07
**Age (years)**	-	-	-	-	-	< 0.01*	-	-
< 25	40	26.7	110	73.3	150	-	1.00	-
25–34	71	19.4	296	80.7	367	-	1.06	0.95–1.17
35–44	51	16.4	260	83.6	311	-	1.10	0.99–1.21
45–54	26	9.8	240	90.2	266	-	1.20	1.09–1.33
≥ 55	12	9.8	110	90.2	122	-	1.17	1.05–1.30
**Location**	-	-	-	-	-	0.55	-	-
Urban	195	16.3	1001	83.7	1196	-	1.00	-
Peri-urban	5	25.0	15	75.0	20	-	0.91	0.70–1.18
**Job designation**	-	-	-	-	-	-	-	-
Pharmaceutical personnel	19	22.9	64	77.1	83	< 0.01*	1.00	-
Physicians	34	12.8	232	87.2	266	-	1.15	1.02–1.31
Nursing and midwifery	45	12.9	305	87.1	350	-	1.12	0.99–1.27
Laboratory health worker	78	31.1	173	68.9	251	-	0.92	0.80–1.06
Other health workers	11	9.8	101	90.2	112	-	1.20	1.05–1.36
Community support	13	8.4	141	91.6	154	-	1.20	1.06–1.36
**Facility ownership**	-	-	-	-	-	< 0.01*	-	-
Public	191	17.7	889	82.3	1080	-	1.00	-
Private	9	6.6	127	93.4	136	**-**	1.15	1.08–1.22

**Total**	**200**	**16.4**	**1016**	**83.6**	**1216**	**-**	**-**	**-**

Note: *N* of 1216 as against 1227 excludes HCWs who had unknown vaccination status. Job designation ‘Other HCWs’ include hospital cleaners, ward maids, radiographers and so on.

HCW, healthcare workers; CI, confidence interval.

* , Represent statistically significant variables at 0.05.

### Participants’ confidence in the effectiveness of the COVID-19 vaccines

A total of 1025 (83.5%) of the 1227 HCWs strongly agreed that being vaccinated against infectious diseases such as measles and tuberculosis reduces the risk of a person getting sick or dying. While only 760 (61.9%) strongly agreed that vaccination against COVID-19 reduces the risk of a person getting sick or dying (Figure 2b).

### Willingness to recommend COVID-2019 vaccination

A total of 1135 HCWs (92.4%) expressed willingness to ‘Definitely/Probably Recommend’ vaccination, with more female (93.6%) HCWs being willing. A large proportion of HCWs working in public and private healthcare facilities, 91.9% and 97.1%, respectively, favoured recommending COVID-19 vaccination, although those in private facilities are more willing than those in public facilities (*p* = 0.03). There was no difference in willingness to recommend based on age, location and profession of HCWs (Table 1).

### Eagerness to recommend COVID-19 vaccination among vaccinated and unvaccinated healthcare workers

The study showed that HCWs who were vaccinated against COVID-19 were more likely to recommend the vaccine compared to those who were not vaccinated (*p* = 0.0001). Among the unvaccinated, 24.0% were not willing to recommend the COVID-19 vaccine, while only 4% of the vaccinated expressed unwillingness to recommend it (Table 3).

**TABLE 3:** Willingness to recommend vaccination based on vaccination status of healthcare workers.

Responses	HCWs who have received at least one dose of the COVID-19 vaccine				Total	
	Unvaccinated		Vaccinated			
	*n*	%	*n*	%	*n*	%
Definitely/probably recommend	152	76.0	975	96.0	1127	92.7
Not recommend vaccination	48	24.0	41	4.0	89	7.3

**Total**	**200**	**100**	**1016**	**100**	**1216**	**100**

Note: *p* = 0.0001.

HCW, healthcare workers; COVID-19, coronavirus disease 2019.

### Willingness to recommend COVID-19 boosters based on source of information

Most HCWs expressed willingness to recommend the COVID-19 booster doses if the recommendation was by the WHO (62%). In contrast, only 23.2% indicated that they would do the same if the recommendations were from the Federal Ministry of Health (Figure 2c).

### Reasons for vaccine hesitancy

Furthermore, 90% of the 200 health workers who were not vaccinated at the time of this study mentioned the following as reasons for their hesitancy: (1) The belief that the vaccine development and authorisation were rushed (26.1%), (2) concerns about serious side effects (17.8%), (3) not having enough information about the vaccine to make a decision (11.7%), (4), optimism that there will be other treatment for COVID-19 (11.7%), (5) feeling of not being at risk of infection of COVID-19 (10.6%), (6) feeling of not being at risk of getting very sick or dying from COVID-19 (8.9%), (7) concerns about conspiracies including microchips (4.4%), (8) the belief that vaccines can cause the disease that they are designed to protect against (3.3%), (9) someone in family or community preventing vaccination (1.1%), (10) perceived invulnerability to reinfection following recovery from the past COVID-19 infection (1.1%), (11) people in community being fearful of those who have been vaccinated (1.1%), (12) preferring different vaccine than the ones available in the country (1.1%), (13) perceived feeling that the vaccines available in the country are not protective (0.56%) and finally, (14) vaccines being against an individual’s religious belief (0.56%).

## Discussion

This study showed a high rate of COVID-19 vaccine uptake among HCWs in Ibadan, with approximately three-quarters of the participants being fully vaccinated and a few having received a part of their respective vaccine types. The vaccine uptake rate in our study was higher than acceptance rates reported among adult populations in some states in Nigeria, which ranged from 26.0% to 74.5% in 2021.^23^ As of 2021, data from various African countries show that the estimated acceptance rates for COVID-19 vaccines are 37% in North Africa, 28% in Central Africa, 48% in West Africa, 49% in East Africa and 90% in Southern Africa among the general population. The rate of vaccine acceptance in this study is also higher than the estimated pooled vaccine acceptance rate of 48% reported among HCWs drawn from several African countries.^24^ One possible reason for the variation in vaccine acceptance rates between our study and others could be the timing of the research. The studies referenced in Ackah et al., 2022 were conducted in 2021 and early 2022, shortly after the COVID-19 vaccine was introduced when there was limited knowledge about its effectiveness and safety. In contrast, our study was conducted in May 2023. The findings of this study show that HCWs’ acceptance and uptake of the vaccine improved over time. It also suggests that efforts to promote the vaccine were effective and that increased knowledge about the COVID-19 vaccine, as the pandemic continued, may have also led to a more positive attitude towards the vaccine among HCWs.^25^ However, because of the crucial role HCWs play in promoting vaccine acceptance within the community, it is essential to target educational interventions specifically at unvaccinated HCWs to improve their perceptions and attitudes towards the COVID-19 vaccine.

Most HCWs in our study reported easy access to the vaccine, indicating the effectiveness of strategies deployed by government agencies responsible for vaccine distribution. This level of accessibility may have contributed to the high level of uptake of the vaccine. The prioritisation of HCWs during the initial vaccination rollout facilitated easier access to vaccines, as they were directly provided to most health facilities. Furthermore, the study conducted in urban health facilities may have played a role in improving vaccine accessibility in the surveyed health facilities. Other studies have also shown that enhanced vaccine accessibility leads to higher vaccination uptake rates among HCWs.^14^

About half of the HCWs in Ibadan reported that they were not confident in answering COVID-19-related questions. Healthcare workers are typically considered a reliable source of information, but our findings indicate a lack of confidence in addressing important issues related to the virus. This finding is of great concern as accurate information is crucial in disease prevention and management.^12,14^ The widespread use of smartphones and social media has led to the dissemination of unverified news and propaganda, potentially influencing HCWs’ knowledge and confidence levels regarding vaccines and other related topics.^26^ It is thus imperative to improve health education among HCWs in Ibadan to ensure they are equipped with accurate and up-to-date information.

The high vaccine uptake among older age groups may be attributed to policies that prioritised vaccinating the elderly because of their increased vulnerability to severe outcomes of COVID-19. Evidence during the pandemic indicated that older age groups experienced more severe cases of COVID-19.^27^ In contrast, younger HCWs may feel invulnerable to the virus and may be influenced by myths and misinformation circulating on social media. A study conducted among the general population in three geopolitical zones in Nigeria before introducing COVID-19 vaccines showed the highest infection rate among asymptomatic younger participants.^28^ Targeted campaigns tailored to the unique circumstances of younger HCWs could help increase vaccine acceptance among this group. This observation is consistent with those reported among HCWs in several other studies.^29,30,31,32^

Healthcare workers working in laboratories and pharmaceutical roles had lower vaccine uptake, possibly because of their perceived lower risk of exposure to COVID-19 patients because they do not interact with patients directly compared to other categories of HCWs. Furthermore, their job roles may have also made them more familiar with the traditional process of vaccine production and preapproval safety trials timeline, which is usually longer; thus, they may be more apprehensive than other categories of HCWs who are not too concerned with the details involved in vaccine production. A comprehensive educational approach to build trust in science and address vaccine safety and efficacy concerns may help reduce hesitancy among this group.^29,31^ Community support workers, on the other hand, had the highest vaccination acceptance, likely because of their direct involvement in administering the vaccines. Furthermore, a higher vaccination rate was found among HCWs who worked in private health facilities compared to their counterparts in the public sector. This may be because of the degree of enforcement of procedures and instructions in private health facilities. However, enforcement issues need to be approached with caution as efforts to enforce strict adherence in some public facilities in the country have resulted in protests and legal redress as people are more conscious of their rights, especially with issues regarding their health. The higher rate of uptake by HCWs in primary health facilities might be because they were more directly involved in vaccine administration. In contrast, those working in secondary and tertiary facilities are often involved in patient care. In addition, those in higher levels of healthcare are most likely to raise questions about the speed of the development and approval for the use of the vaccine. The results from this study is consistent with findings from other studies, where negative emotional responses, including anxiety, doubts, fear and worry, were found to be drivers of vaccine hesitancy.^31^

Many HCWs in Ibadan strongly agreed that vaccines protect against other infectious diseases, such as polio, measles and tuberculosis, with which they were familiar. However, fewer held the same view regarding the protective capacity of the COVID-19 vaccine. This finding may be because HCWs have seen cases of vaccinated individuals who still became sick or died of COVID-19, unlike other situations where vaccination provided long-lasting protection against infection and diseases. Providing more information to HCWs in Ibadan about the safety and efficacy of the vaccine may help improve their trust.^14^

Female HCWs in Ibadan showed more willingness to recommend the vaccine than their male counterparts, and this may be attributed to the fact that women are more predisposed to accept and recommend vaccinations because of their role in presenting their babies for vaccination and being sensitised about the importance of vaccines. However, other studies have reported that women expressed lower levels of agreement that vaccines are safe and effective and, thus, less likely to recommend them.^33^ In this study, no difference was found in willingness to accept the vaccine between males and females. However, results from an international survey among low- and middle-income countries showed that the literature on gender and COVID-19 vaccine acceptance is mixed, with most studies indicating higher male acceptance. However, higher female acceptance has been reported in another study conducted across 19 countries.^34^

Willingness to recommend vaccination was highly associated with positive vaccination status. This is expected because HCWs are more likely to promote vaccination to others if they have been vaccinated themselves. Researchers in some other countries have reported similar findings.^13,14^ A significant finding is that the majority of HCWs were more inclined to recommend a COVID-19 booster dose if the guidance came from the WHO rather than the country’s Ministry of Health. Although this finding may suggest a potential distrust in the national government,^35^ it is important to observe that even most Ministry of Health in Africa refer to information from WHO. This may have influenced the preferences of the participants. This observation is not unique to Nigeria, as similar trends have been reported in many other developing countries.^36^

Many HCWs who declined to receive the vaccine expressed fear that the vaccine development was rushed as their primary reason for hesitance, followed by concerns about serious side effects and a lack of information to make an informed decision about the vaccine. These findings align with other studies indicating that concerns about vaccine safety, effectiveness and the rapid development of COVID-19 vaccines have contributed to vaccine hesitancy.^23^

This study is not without its limitations. One major limitation is the use of purposive sampling, which may limit the generalisability of the findings. Efforts were not made to have equal representation of the various categories of study participants, which we compared during this study. It is particularly necessary to consider this limitation when drawing conclusions from our comparison of vaccine acceptance across different age groups, categories of HCWs and facility types, even in cases where statistically significant findings were observed, these results still need to be interpreted with caution. In addition, there is the possibility of recall bias among participants as most of them received their vaccines at a distant time. This may have affected their ability to remember all the details surrounding the vaccination process, potentially leading to inadvertent inaccuracies in their response during questionnaire administration. Additionally, we did not evaluate the level of knowledge of the HCWs about COVID-19 before asking them to provide responses regarding their confidence in addressing COVID-19-related questions. The absence of specific questions about role of the Nigeria Centre for Disease Control in booster vaccine recommendations may also have impacted our findings on willingness to recommend vaccination in Nigeria. Another limitation was that this study was conducted in an urban area and the outcome may not fully reflect the realities among HCWs working in healthcare facilities in rural areas. Finally, it is worthy of note that HCWs’ perception of the vaccine may have changed since the early phases of vaccine availability, potentially leading to the higher acceptance rate observed in this study.

## Conclusion

Our study showed a high rate of COVID-19 vaccine uptake among HCWs in Ibadan, Southwest Nigeria. The convenience of access to the vaccine enhanced vaccine uptake, and the majority were willing to recommend the vaccine to others. Healthcare workers in Ibadan trusted recommendations from the WHO more than those from the Ministry of Health. The barriers to vaccine acceptance included concerns about the fast development and approval of COVID-19 vaccines, as well as fears of serious side effects. Therefore, it is important to improve education and awareness among HCWs about the COVID-19 vaccine to address safety concerns and provide clear information from reliable sources such as the WHO website.
